# Putting the brakes on chondrosarcoma

**DOI:** 10.18632/oncotarget.5242

**Published:** 2015-08-22

**Authors:** Pavel Krejci

**Affiliations:** Department of Biology, Faculty of Medicine, Masaryk University and International Clinical Research Center, St. Anne's University Hospital, Brno, Czech Republic

**Keywords:** Chromosome Section, chondrosarcoma, FGFR3, cartilage, growth, inhibition

Primary cartilaginous tumors often arise from endochondral ossification, range from benign endochondroma and osteochondroma to malignant chondrosarcoma, and are notoriously resistant to chemotherapy or radiation. This urges development of novel therapeutic approaches particularly in chondrosarcoma, which is a terminal disease in more than 90% of unresectable cases [1].

Now research of Zhou and colleagues [2] suggests an intriguing possibility that activation of mechanisms that naturally restrict the skeletal development may have a therapeutic potential in chondrosarcoma. Authors show that chondrocyte-specific deletion of gene encoding fibroblast growth factor receptor 3 (*Fgfr3*) induces multiple tumor lesions adjacent to growth plate cartilage in mice. These lesions are characteristic of endochondromas and osteochondromas, and show elevated expression of chondrocyte mitogen and morphogen, the indian hedgehog (IHH). Chemical inhibition of IHH signaling ameliorated endochondroma development, demonstrating that FGFR3 acts as a cartilage tumor suppressor which exerts its function via negative control of IHH production [2].

FGFR3 is a receptor tyrosine kinase which transduces the extracellular communication signals delivered by members of FGF family of growth factors. In its major physiological function, the FGFR3 acts as a ‘brake’ on bone growth, as proven by skeletal overgrowth in *Fgfr3* null mice, or in human camptodactyly, tall stature, and hearing loss (CATSHL) syndrome, caused by loss-of-function mutations in *FGFR3*. On the other hand, germline activating mutations in FGFR3 trigger profound inhibition of chondrocyte proliferation, resulting in severe or even lethal human skeletal dysplasias, such as achondroplasia and thanatophoric dysplasia [3, 4]. Interestingly, the excessive FGFR3 activation in skeletal dysplasia targets several pathways known to be dysregulated in chondrosarcoma, such as chondrocyte growth factors IHH and parathyroid hormone-related protein (PTHrP), the component of extracellular matrix collagen type 2, and cell cycle regulators CDK4 and CDK6 [1, 4, 5].

Altogether, the abovementioned evidence suggests that the therapeutic effect of FGFR3 activation in chondrosarcoma is worth evaluation. To determine whether the chondrosarcoma growth may be attenuated by FGFR3, we first need to establish if the chondrosarcoma cells retain their responsiveness to the FGFR3 activation. Although early studies demonstrate that FGFR3 activation causes potent growth-arrest in cultivated chondrosarcoma cells [6], *in vivo* evidence is necessary to confirm these data. Crossing of animals carrying mildly activating FGFR3 mutation, such as G380R which is typical for achondroplasia, with established transgenic mice models to chondrosarcoma should confirm the inhibitory effect of FGFR3 activation on chondrosarcoma growth *in vivo*. Subsequently, a feasible mode of FGFR3 activation in the chondrosarcoma lesions needs to be found. This should not pose a significant problem, since growth plate FGFR3 may be activated via administration of FGF ligand, FGF2, as shown in both limb explant cultures as well as transgenic mice engineered to overexpress FGF2 [4, 7]. In addition, the *in vivo* FGF2 administration appears to be safe, according to studies evaluating FGF2 effect on periodontal regeneration [8].

## References

[1] Bovée JV (2010). Nat Rev Cancer.

[2] Zhou S (2015). PLoS Genet.

[3] Toydemir RM (2006). Am J Hum.Genet.

[4] Foldynova-Trantirkova S (2012). Hum Mutat.

[5] Tarpey PS (2013). Nat Genet.

[6] Aikawa T (2001). J Biol Chem.

[7] Coffin JD (1995). Mol Biol Cell.

[8] Kitamura M (2011). J Dent Res.

